# Role of Phosphodiesterase in the Biology and Pathology of Diabetes

**DOI:** 10.3390/ijms21218244

**Published:** 2020-11-03

**Authors:** Agnieszka Kilanowska, Agnieszka Ziółkowska

**Affiliations:** Department of Anatomy and Histology, Collegium Medicum, University of Zielona Gora, Zyty 28, 65-046 Zielona Gora, Poland; a.kilanowska@cm.uz.zgora.pl

**Keywords:** phosphodiesterases, insulin secretion, cAMP, cGMP, diabetes mellitus

## Abstract

Glucose metabolism is the initiator of a large number of molecular secretory processes in β cells. Cyclic nucleotides as a second messenger are the main physiological regulators of these processes and are functionally divided into compartments in pancreatic cells. Their intracellular concentration is limited by hydrolysis led by one or more phosphodiesterase (PDE) isoenzymes. Literature data confirmed multiple expressions of PDEs subtypes, but the specific roles of each in pancreatic β-cell function, particularly in humans, are still unclear. Isoforms present in the pancreas are also found in various tissues of the body. Normoglycemia and its strict control are supported by the appropriate release of insulin from the pancreas and the action of insulin in peripheral tissues, including processes related to homeostasis, the regulation of which is based on the PDE- cyclic AMP (cAMP) signaling pathway. The challenge in developing a therapeutic solution based on GSIS (glucose-stimulated insulin secretion) enhancers targeted at PDEs is the selective inhibition of their activity only within β cells. Undeniably, PDEs inhibitors have therapeutic potential, but some of them are burdened with certain adverse effects. Therefore, the chance to use knowledge in this field for diabetes treatment has been postulated for a long time.

## 1. Introduction

Glucose is the major physiological stimulator of insulin secretion from pancreatic β cells. The first and second phases of insulin release are quantitatively dependent on the enhancement of the metabolic pathway by approximately 50% [1,2], and its increase in plasma leads to a significant rise in glucose uptake and metabolism in pancreatic β cells. In consequence, an increase in adenosine triphosphate/adenosine diphosphate (ATP/ADP) ratio is observed, and it leads to a closure of ATP-sensitive K^+^ channels (K_ATP_ channels). The cascade signal results in plasma membrane depolarization, opening of voltage-gated plasmalemic Ca^2+^ channels (VGCC), and the influx of calcium ions [3,4]. The consequence of these occurrences is an increase in intracellular calcium concentration ([Ca^2+^]_i_) resulting from the inflow through plasmalemmal voltage-gated calcium channels. The effect of which is exocytosis of insulin granules [4,5]. It was also observed that exocytosis of insulin is stimulated by an increases in cAMP [6,7].

## 2. The Second Messengers cAMP and cGMP—The Roles in Pancreas

Cyclic nucleotides are responsible for regulation of many essential to physiological and pathophysiological processes. cAMP is involved in the regulation of energy metabolism, many processes depend on its presence and concentration, including the rate of triglyceride lipolysis, and cAMP signaling pathways also modulate gluconeogenesis, glycogenolysis, and thermogenesis [8]. The complexity and specificity of the cAMP action depend on the spatial localization and temporal dynamics of cell signaling [9]. Undeniably, cAMP is an intracellular enhancer of insulin secretion stimulation due to glucose [10,11]. It was identified that there is a strong interdependence between cAMP and Ca^2+^ affective performance of adenylyl cyclases (ACs) isoforms. An intracellular increase in cAMP concentration affects transmembrane AC via Gs-coupled receptors [12]. The research has shown expression in pancreas islets of all nine isoforms of adenylyl cyclases [13,14]. cAMP also stimulates exocytosis of insulin in a protein kinease A (PKA)-independent manner [14]. Signal transduction mechanisms usually result from ligand binding. The first recognized effect is the activation of seven-transmembrane G protein-coupled receptors (GPCRs) and stimulation of the Gs, which consistently increase the ratio of guanosine diphosphate/guanosine-5’-triphosphate (GDP/GTP) [15]. Moreover, the stimulation of anchored into membrane adenylyl cyclases (ACs) is observed by Gs protein. Conversion of ATP to cAMP increases the efficiency of two cAMP receptors: PKA or EPAC (exchange protein directly activated by cAMP), but, in addition, it also generates tissue-specific phosphodiesterase (PDE) activity [16]. An increase in cAMP causes dissociation of PKA active catalytic subunits and the dependent phosphorylation of various proteins that affect metabolism, proliferation, integrin-metiated cell adhesion, cell–cell junction formation, exocytosis, and insulin secretion. Nucleotides are thought to act mainly at many levels via PKA [11] and EPAC by generating Ca^2+^, and, in consequence, insulin secretion [17]. It also does not exclude PKA-independent interaction, initiating secretion on its own [10]. Specifically, cAMP plays a key role in hormonal modulation of insulin secretion because basal cAMP levels appearance is necessary for glucose to induce insulin secretion [18]. The islet cyclic AMP is increased in response to glucose, and agents that elevate islet cyclic AMP augment glucose-induced insulin secretion [4,19].

On the other hand, the role of cyclic guanosine monophosphate (cGMP) as a nucleotide modeling the process of insulin secretion and glucose metabolism has not yet been fully explained. The cGMP pathway in β cells appears to regulate glucose metabolism through the upstream activation of NO [20]. The guanylate cyclase (GC) act as membrane-bound proteins controlled by extracellular ligand activation and catalyzes the synthesis of cGMP from GTP. Thus, activation of the GCs results in an increased cGMP concentration [21], which activates PKG and related signal transduction pathways [22]. Moreover, cGMP signaling presents a mechanism of spatial organization similar to cAMP [23]. In contrast, in the pancreatic, β cell is involved in the induction of apoptosis and potentiation of insulin secretion. However, the course of these processes remains largely undefined [21]. It was also suggested that cGMP could have an antiapoptotic effect in β cells; therefore, the control of its cellular concentration may mark a new direction in the treatment of diabetes [24].

## 3. PDEs in Basic Research—GENERAL Outline

The phosphodiesterase family consists of 11 structurally similar isoforms [25]. Expression PDE genes lead to the creation of more than 100 mRNA products and over 50 phosphodiesterase isoforms [26,27,28]. Literature data report that the genes of PDEs contain several transcriptional subunits that determine the structural differences of the resulting proteins [29]. Distinctions between individual isoforms determine many important factors, for instance, structure and different catalytic properties affecting the regulation of specific signaling pathways [30,31]. Critical for catalytic functions that are common to all PDEs are two consensus metal-binding motifs and the histidine-rich sequence [32,33,34]. It was demonstrated as three functional domains in all subtypes: a catalytic core that is conserved for all PDE types and two regulatory regions: N- and carboxyl terminus [35,36]. The C-terminus is similar for all PDEs (not applicable to PDE6) and has 30% sequences identified between families. Characteristic for each enzyme is the N-terminus of regulatory domains. The presence of a structural domain and specific amino acid sequence determines the location in a cell, cell organelle, or membrane. It contains regulatory sequence motifs and has a cofactor or cGMP-binding site [23]. Moreover, domains present in regulatory regions may be subject to various modifications, for example, phosphorylation, binding sites for allosteric ligands, or selective effectors [37]. The individuality of the N-terminal regulatory region for each of the PDEs is a tool and it can be directly used in therapeutic projects for targeted drug action. The PDE inhibitors are a large group of biologically active compounds and they can be used to treat many disorders [25,26,38]. Nevertheless, their use is hampered by their broad expression in a variety of tissues [39,40].

For years, it has been debated which isoforms are present in pancreatic β cells and what role they play in the process of insulin secretion. Many scientific publications emphasize that selective pharmacological inhibition of PDEs directed against β cells may be the key to the treatment of diabetes, especially type 2 [9,11,25,41]. It has been previously shown that the role of all PDEs in the pancreas was mainly limited to cAMP/cGMP hydrolysis and its inhibition induced cAMP/cGMP accumulation [41] and has insulin secretagogue activity [10,11]. Indeed, PDEs present in pancreatic β cells showed many differences, ranging from the location in the cell through the timing, regulation and duration of each or cAMP/cGMP specificity, etc. Although, a detailed understanding of the action mechanism in individual PDE subtypes in the insulin secretion process is still unclear. The PDE1, PDE3, and PDE4 families are widely considered to be important for the regulation of cytosolic cAMP levels and glucose-induced insulin secretion from pancreatic islets. Among all the studies focused on the role of PDE in the context of insulin secretion and β-cell metabolism, these enzymes are undoubtedly superior to other enzymes.

### 3.1. The PDE1 Family

The PDE1 family is encoded in humans by three genes: *2q32* for PDE1A, *12q13* for PDE1B, and *7p14* for PDE1C [40]. Genes for PDE1 subtypes have alternatives promoters, thus creating a multitude of proteins by alternative splicing. Subtypes of PDE1 (three subtypes PDE1A–PDE1C) are widely expressed in human tissues. The molecular weight of the protein is varied and contains 58 to 86 kDa per monomer [42]. It was recognized that regulatory domains of PDE1 have two N- terminal regulatory domains that contain an Ca^2+^/CaM binding region and a C- terminal helical bundle [31]. Two N-domains are separated by the autoinhibitory region of the catalytic domain. The PDE1 activation process involves Ca^2+^ binding to the free CaM region, which results in the activation of the Ca^2+^-CAM and changes in the conformation of the PDE1 structure (Ca^2+^-CaM*-PDE1). The PDE1 subtypes were phosphoryleted by different kinases. PDE1A izoensymes are substrates of the cAMP-dependent protein kinase PKA, while PDE1B phosphorylation occurs through CaM kinase II in a Ca^2+/^CaM-dependent manner [31].

Individual PDE1 isoenzymes may show slight differences in kinetics, phosphorylation, or dephosphorylation and specific sensitivity to inhibition by various pharmacological factors and are considered to be important in the context of the crosstalk mechanism between the cyclic nucleotide and calcium [43]. Nevertheless, it is suggested that the PDE1 family has low basal activity; increasing intracellular cAMP and Ca^2+^ ions increases their activity. It should also be emphasized that these enzymes exhibit dual specificity for the hydrolysis of both cAMP and cGMP but with different affinities [33]. The presence of PDE1 has been demonstrated in the brain, heart, lung, smooth muscles, testis, and olfactory epithelium. The activity of PDE1 was observed in the cytosol and in the cell nucleus, which indicates the regulation of its action by transcription factors [31,42]. It has been predicted that PDE1 may play a key role in pancreatic β cells in terms of Ca^2+^-dependent signaling pathways [44], which are strongly associated with insulin secretion. These assumptions were also confirmed in the first reports, which were carried out on isolated pancreatic islets from the rats. The results showed the activity (dependent on intracellular concentration of Ca^2+^ and cAMP) and changes in insulin secretion following PDE1 inhibition [45]. Similar studies also confirmed the role of PDE1 in the insulin secretion process and its dependence on the presence of Ca^2+^ and cAMP and were also carried out by Lipson and Oldham [46] and Capito et al. [47]. The use of a selective PDE1 inhibitor (vinpocetine) turned out to be ineffective in increasing insulin secretion (rat pancreatic islets), despite a decrease in PDE1 activity. However, inhibitor dose-depend insulin secretion from human pancreatic islets was observed [48]. Han et al. [49] showed the presence of mRNA for PDE1 isoforms in the pancreas, which was confirmed by Waddleton et al. [41]. While Dov et al. [39] confirmed the weak expression of PDE1B but not of PDE1C and PDE1A in INS1-E cells. It is considered that the PDE1C isoform is the most strongly expressed in mouse cell lines [39]. The effects of selective inhibition of 8MM-IBMX and increased insulin secretion from isolated mouse pancreatic islets was also presented. The obtained results of this experiment presented the same efficiency as was obtained after the action of milrinone (the selective PDE3 inhibitor). The regulatory influence of PDE1C on the process of insulin release from βCT3 cells and pancreatic islets was detected [49]. However, it should be emphasized that the observed effects may have been directly the result of the superphysiological glucose concentration used in the experiments, which rather confirms that PDE1 activity is only revealed in conditions of high concentration of cAMP and Ca^2+^ on which its action depends. Nevertheless, subsequent studies conducted by Waddleton et al. [41] detected the expression of PDE1C in rats islets and INS-1 (832/13) cells. Transfected INS-1 cells (832/13) also revealed a high increase in insulin secretion (2–3 times) **via** electroporation, especially in response to siPDE1C. In other studies, significant stimulation of insulin secretion by 8MM-IBMX and its effect on resting cAMP concentration in INS-1 cells indicating the advantage in these cells of the participation of PDE1 in the mechanisms associated with insulin secretion over the PDE3 and 4 isoforms have been presented [13]. Whereas, in studies carried out on MIN6 cells, an increase in insulin secretion was observed in the presence of 20 mM glucose and a selective inhibitor MM-IBMX for PDE1 [9]. The above observations have not been confirmed on the cell line BRIN-BD11 [50]. As previously mentioned, the mRNA of PDE1 was shown in the human pancreas, too. In addition, activity (about 30%) of these isoforms was determined from homogenized human pancreatic islets [25]. Selective PDE1 inhibition has been shown to increase glucose-induced insulin secretion [13,25,51]. The upregulation of PDE1 after high glucose concentrations on which its action depends is considered to be dependent on human β cells. Moreover, the participation of this isoform may protect β cells against the toxic effects of excess fatty acids. It has been previously noted that mRNAs of PDE1B-C are most expressed in human pancreatic islets and are the most effective isoform for GSIS [13] but the mechanism is still not fully understood [31].

### 3.2. The PDE3 Family

The PDE3 family is encoded by two distinct genes. The genes for PDE3A and PDE3B are located on chromosomes *12p12* and *11p15.1*, respectively. Expression for three variants has been described for PDE3A: PDE3A1 (molecular mass 136 kDa), PDE3A2 (molecular mass 118 kDa) and PDE3A3 (molecular mass 94 kDa). For PDE3B, no splice variant or alternative start sequence has yet been identified, but different sizes of proteins have already been reported. The cDNA (PDE3A and PD3B) is similar and contains a C-terminal catalytic region and two N-terminal regions which reveals function in many intracellular processes. The regulatory regions appear in both the intracellular membrane and cytosol. The activation sites for PKA (protein kinase A) and PKB (protein kinase B) are located between N- terminal regions [19,42,52]. The family of PDE3 hydrolyzes both cAMP and cGMP, but the rate of hydrolysis for cAMP is about 4–10 fold greater than for cGMP. However, cGMP is recognized as a competitive inhibitor for cAMP hydrolysis [31,42,43]. Although the affinity for cGMP is significantly higher and is responsible for the cAMP inhibition of hydrolysis.

It has already been well documented that strong PDE3A expression is a characteristic of the cardiovascular system, whereas the PDE3B isoform is often expressed in tissues associated with the regulation of glucose and lipid metabolism [30]. In addition, the PDE3 family consisting of two enzymes distributed in tissues, particularly important for energy homeostasis [10]. Although confirmation of the presence of both isoforms in β cells of the pancreatic islets was observed [7,50,51,53], the isoform of PDE3B is considered more significant in the context of β cell function [39,41,54,55,56]. Many animal experiments have clearly shown that PDE3B is important for the metabolism of liver, adipocytes, pancreatic β cells and thus plays an important role in the regulation of energy homeostasis [57,58,59]. Therefore, it is widely recognized as crucial for the process of insulin secretion (regardless of species) from all forms of PDEs determined to be present in the pancreas. The relationship between the membrane or cytosol location of the enzyme and the effect on the insulin exocytosis machinery is also significant in terms of the efficiency of the inhibition process [10]. PDE3 isoforms have been observed to be activated in response to cAMP enhancers as well as insulin, insulin-like growth factor 1 (IGF-1), leptin via phosphatidylinositol 3-kinase (PIK3)-dependent signals, and IL-4 [33] but also to factors that increase cAMP. The inhibition of PDE3B increases insulin secretion from β cell lines [41], in rodents [51,60,61] and human islet has been confirmed [48,62]. Studies carried out on isolated pancreatic islets of rats confirmed that selective inhibition significantly increases the insulinotropic action of physiological glucose concentration. Incubation of human pancreatic islets with milrinone increased insulin secretion induced by glucose (about 60%). Analysis of PDE activity showed that a decrease in PDE activity was dependent on the concentration of inhibitor. In incubated rat pancreatic islets, a significant (3-fold) elevation of insulin release was found in the presence of milrinone and glucose (8 mM) in comparison with glucose alone. Inhibition efficiency relative to total PDE activity was slightly higher than in human tissue [48]. Amrinone as a selective PDE3B inhibitor also caused higher insulin secretion from rat islets (static incubation in the presence of 6.7 mM glucose), but no effect in insulin secretory response to glucose 16.7 and 30 mM was observed [60] even in the presence of dibutyryl cAMP (DB-cAMP) [61], while trequinsin caused the opposite effect [41]. Pimobendan and 8.3 mM glucose also resulted in increased insulin secretion [63] as well SK&F94836, Org9935, SK&F94120, and ICI118233 augmented insulin release from rats islets in the presence of 10 mM glucose, but this effect was not observed at a 3.0 mM glucose concentration [51], which was also confirmed in another experiment [61]. The non-stimulating glucose concentration and PDE3B inhibitor were ineffective in insulin release. More interesting in this context is the observed insulinotropic effect of succinate enhanced by the inhibition of PDE3B-amrinone. Increased insulin secretion from isolated pancreatic islets resulted from succinate metabolism [60].

Pancreatic perfusion in situ with amrinone and physiological glucose showed insulinotropic effects. The response of the perfused pancreas and its sensitivity to the PDE3B inhibitor was revealed by an immediate increase in insulin secretion [60]. The perifusion data allowed the observation of the effects of the inotropic drug pimobendan in the presence of glucose at a concentration of 16.7 mM [64]. Selective inhibition of PDE3 and GSIS increased 2-fold insulin release compared to control was confirmed [41]. A similar effect was observed in vivo experiments performed on *ob/ob* mice, where the insulinotropic effect and better glucose tolerance were observed due to selective milrinone inhibition [65]. Overexpression (2–3 fold) of PDE3B and special diet (HFD) in RIP-PDE3B/2 mouse contributed to hyperglycemia, pancreatic dysfunction of islets, intolerance of glucose, and insulin resistance, which developed suddenly and blunted insulin secretion [10]. In vivo studies in rats showed that intravenous injection of milrinone at a dose of 1, 5, or 25 μmol/kg resulted in an immediate increase in the concentration of FFA (free fatty acids) and insulin in the plasma. The increase in glucose was only noted in response to 5 and 25 µmol/kg doses. The intravenous injection of milrinone during euglycemic–hyperinsulinemic clamps abolished insulin suppression for both lipolysis and endogenous glucose production while not affecting insulin-stimulated glucose uptake [66].

The studies in vivo and in vitro showed that overexpression of PDE3B in pancreatic β cells abolished the increase in cAMP accumulation and insulin secretion mediated by glucose and glucagon-like peptide-1 (GLP-1) [7,10]. Selective inhibition (milrinone) reduced PDE3 activity in fractionated soluble βTC3 cell lines, and the value oscillated around 70% but was not effective in insulin secretion in the presence of 16.7 mM glucose. Incubated mouse pancreatic islets reacted differently under these conditions, revealing an increase in insulin secretion (about 2-fold). It indicates the differences in the distribution, the effectiveness of individual isoforms, and the complexity of signal transfer functions or pathways in which PDEs occur in natural islets and cells imitating β-cell function [49]. It was also noted that Org 9935 and siguazodan caused a strong inhibitory effect on PDE3 activity (30–40%) in BRIN-BD11 cells, which augmented insulin secretion [50]. Other cell line studies (INS-1 (832/1)) have shown an increased GSIS and trequinsin inhibition [41]. However, in other studies, it was shown that PDE3 in INS-1 cells neither regulates glucose-stimulated cAMP nor increases GSIS and is less efficient than PDE1 in stimulating glucose-induced insulin secretion in humans [13]. Different results were presented by Parker et al. [48]. It has been shown that inhibition of PDE3 is more effective for GSIS in human pancreatic than the other isoforms. The discrepancy could be the effect of biological variability of the donor (general health, BMI, age, hemoglobin A1C, cause of death), lack of information [48], or the conditions of the experiments (for example, islet incubation and pre-incubation times).

### 3.3. The PDE4 Family

The PDE4 family in humans consists of over 25 members characterized by differentiation of cellular localizations and molecular targets [40]. To date, four genes have been identified on three different human chromosomes. The cytogenetic localization for each of the PDE4 isoforms is as follows: PDE4A-*19p13.2-q12*, PDE4B-1p31, PDE4C-*19p13.*1, and PDE4D-*5q12* [67]. The complexity of the protein (from 50 to 125 kDa) is due to unique promoters and alternative mRNA splice variants generated from each gene, and the resulting short and long splice variants assemble a functional protein [68]. The differences for each isoform in their N-terminal regions [69] were shown to include sequences responsible for subcellular locations or signalosomes. The involvement in the modulation of the response to signals from regulatory molecules was also presented [70]. Moreover, a specific sequence motif was observed in the N-terminal region, which, depending on the PDE4 enzyme categories (especially for long, short, super-short forms), usually contain two blocks (conserved regions 1 and 2- up-stream conserved region (UCR)1 and UCR2) [71]. The long splice variants of PDE4 contain UCR1 as well as UCR2, the short splice variant only UCR2. The super-short form contains a truncated UCR2, and the dead-short isoform lacks this portion [70]. The UCR1 and UCR2 sequences are important with respect to PDE4 functions as they may be involved in the feedback regulation system, especially with reference to phosphorylation by PKA or extracellular signal-regulated kinases (ERK) [72,73]. Oligomerization enhanced the membrane association for both the long and short forms through UCR1 and UCR2 [74]. Within the UCR1 present in the long variant, the site of PKA phosphorylation is found. The phosphorylation process lowers the ability of UCR1 to interact with UCR2 and is associated with an increase in PDE4 activity, and, consequently, the activation of cAMP hydrolysis [72]. For some isoforms, the inhibitory phosphorylation by Mitogen-activated protein kinases (p42^MAPK^) and the ERK2 subunit located in the catalytic domain was detected. It is also interesting from the point of view of functionality to ensure the crosstalk between ERK2 activation with the regulation of cAMP signaling [73]. The activity of the short form of PDE4 is induced by ERK has been noticed, while the long form of the enzyme inhibits its activity. The anchored complex of PKA (A-kinase anchoring proteins (AKAP) and PDE4 should also be mentioned, which not only indicates the subcellular localization (centrosomal and perinuclear) but is also involved in temporal control and termination of the cAMP signaling event [75]. PDE4 provides the major portion of cAMP-hydrolyzing activity [76]. It has been known for a long time that this enzyme is expressed almost ubiquitously [77,78]. Moreover, it was confirmed that it could be involved in major diseases, such as inflammatory or neurological disorders [74]. Studies conducted on the basis anti-inflammatory properties of PDE4 inhibition are currently being explored as therapeutic drugs for the treatment of certain respiratory diseases or in the therapy of psoriasis and depression [40]. It is well documented that PDE4 is expressed in β cells lines and in rodent pancreatic islets [11,25,39,41,48,49,51,56]. The presence of PDE4C in human pancreatic islets was confirmed. The fact that PDE4C was found to be the most important in the human body seems to be significant, while the expression of the PDE4B and PDE4D isoform was found in rats [39]. More isoforms have been detected in INS-1E cell lines: PDE4A, 4B, and 4D [39], but in βTC3 cells only PDE4A and 4D were demonstrated [49]. Some studies carried out on INS-1E cells (832/13) and primary islets of rats indicated that the main PDE-cAMP metabolizing activity is mainly due to the presence of PDE3 and PDE4 [41]. Rolipram, the selective inhibitor, stimulated intracellular cAMP concentration 2–3 fold in βTC3 cells and increased glucose-dependent insulin release (16 mM). A similar effect was also observed after inhibition of PDE4 by RO20–1724 [49]. It has also been shown to increase insulin secretion from BRIN-BD11 cells in the presence of glucose and rolipram [49,50], thus emphasizing the greater sensitivity of PDE4 in cell lines, which was systematically demonstrated in other research. Insulin secretion from INS-1 cells (832/13) was observed due to the presence of selective PDE4 inhibitors: roflumilast and L-826141, and glucose [41]. The effect of PDE4 inhibition (roflumilast and L-826141) on GSIS in INS-1 (832/13) cells augmented a threefold insulin release compared to control [41]. Results have been confirmed by other studies [13]. An increased insulin release was observed in INS-1 cells in the presence of rolipram and glucose 18 mM due to glucose alone. Nevertheless, PDE4 was found to regulate resting cAMP in INS-1 cells, and the additional presence of glucose did not exacerbate this effect [13]. Although other literature data provide contradictory information regarding the specific role of the PDE4 family in insulin secretion. The secretion from isolated pancreatic islets from rats has been shown to have no effect of rolipram and ICI63197 and GSIS [51]. The results of in vitro studies on human pancreatic islets and cells based on selective PDE4 inhibition are also inconclusive. It was shown that rolipram in the presence of 16.7 mM glucose significantly contributed to the intracellular increase in cAMP levels in human β cells. In the presence of glucose 1.7 mM, changes in resting cAMP levels were also shown. The results obtained from the incubation (1h) of isolated human pancreatic islets (4 non-diabetic donors) in the presence of rolipram and glucose 16.7 mM are not conclusive. A positive effect was observed only in two out of four cases [13]. Unfortunately other studies showed no change in secretion despite the use of rolipram (different concentrations) and 2.8 mM and 8.0 mM glucose [48]. The above data ambiguously define the role of PDE4 in the process of insulin secretion. These differences may explain the predisposition of a particular inhibitor, the experimental conditions and/or be a consequence of the material used in the research.

### 3.4. Other PDE

Based on various molecular techniques, the presence of other isoforms in pancreatic islets of humans (PDE7A, PDE8A, PDE10A), rodents (PDE8B), and insulin-secreting cell lines (PDE2A, PDE5A, PDE8A, PDE9A) has been confirmed [25,39]. Moreover, the presence of PDE11A has been confirmed in rat islets and INS-1 cells (832/13) [41]. The variability of the results obtained from different species and cultured cells is still being verified. Nevertheless, it has been established that all labeled phosphodiesterases contribute to determining their overall co-activity in the pancreas. The scope of their functions is also not always clear, and the observations made on them compose a comprehensive picture of the functioning of all forms in the pancreas [4].

### 3.5. The PDE2 Family

The PDE2 family hydrolyzes both cAMP and cGMP nucleotides. The regulatory region of these isoform contains allosteric binding sites for cGMP—named GAF—domains (GAF-cGMP-binding PDEs Anabaena adenylyl cyclase and Escherichia coli Fh1A) [23]. It was recognized that the GAF-A domain is involved in dimerization, and the GAF-B domain is important for cGMP-binding [33]. The GAF-B domain at the N-terminus acts as a negative feedback loop that accelerates the hydrolysis of cyclic nucleotides in the presence of cGMP (similar to PDE5) [31]. Three PDE2 splicing variants have been identified and are expressed in both a variety of cells and tissues, including the brain, heart, liver, lung, adipose tissue, and adrenal gland [79]. Its presence has only been shown in INS-1 cells [39]. The study carried out on isolated rats islets and INS-1 cells revealed that selective inhibition by EHNA and 16 mM glucose did not significantly increase insulin secretion [41].

### 3.6. The PDE5 Family

The PDE5 family specifically hydrolyses cGMP. Two domains GAF-A and GAF-B, within the N-terminal domain, were observed. The GAF-A domain is responsible for cGMP binding, which promotes phosphorylation of PDE5 [80]. Research on the presence in the pancreas shows inconsistent results. Dov et al. [39] did not detect an mRNA signal in rat cattle but confirms the presence of INS-1E in cells. Basic studies conducted in conditions of selective inhibition of the PDE5 (sildenafil) isoform and 16 mM glucose did not significantly increase insulin secretion from rat islets and INS-1 cells. In knockdown experiments in which INS-1 (832/13) cells were transfected with OT-siRNA for PDE5A, any increased insulin secretion in the presence of 8.0 and 16.0 mM glucose was not observed [41]. In animal models, a positive effect of PDE5 inhibition on glucose metabolism and peripheral insulin sensitivity was confirmed. In high-fat-fed mice, a 12-week treatment with chronic inhibition of PDE5 (sildenafil) and L-arginine increased not only cGMP levels but also insulin sensitivity and muscle glucose uptake [81]. Similarly, in rats fed with fructose, an 8-week treatment with sildenafil augmented endothelial function and hyperglycemia after oral glucose load [82].

### 3.7. The PDE7 Family

The PDE7 family hydrolysis cAMP. The regulatory N-terminal domain is still unknown [40].

The immunoblot analysis confirmed molecular results and detected the PDE7A isoform in human islets [25], but the mRNA of PDE7B was not detected in INS-1E [39]. Many publications indicate that PDE7 is susceptible to the effects of the nonselective IBMX inhibitor [4]. To our knowledge, no selective inhibitory action in the context of insulin secretion has been used so far, and the potential mechanism of its effect is still unknown. The first compound reported as a selective inhibitor for PDE7A was IC242, and studies involving theophylline in patients with chronic lymphocytic leukemia B [40,83]. However, two synthesized compounds, GRMS-55 and (±) -LSF, have recently been reported that are of interest for future preclinical and clinical studies related to autoimmune diseases. [38].

### 3.8. The PDE8 Family

The PDE8 family hydrolyzes cAMP and is known to have the highest affinity for cAMP of any PDE [37]. Two domains, REC and Per-Arnt-Sim (PAS), in N-terminal regions of the primary structure have been found [84]. It has been proposed that the PAS domain is involved in protein–protein interactions and PDE8 regulation and is imported for small-molecule ligand binding [85]. Three phosphorylation sites inter alia PKA and PKG have been discovered. Based on many analyses, it has been observed that the caffeine, IBMX, methylxanthines, theophylline are ineffective in PDE8 inhibition [33]. Its presence has been confirmed in the pancreas of humans and mice [9,86]. On this basis, it has been recognized as one of the most important PDEs (next to the basic forms PDE3, 4, and 1) as playing a fairly important role in GSIS [4,13,39]. A limited number of effective pharmacological inhibitor studies have been conducted based on knockdown. Silencing of PDE8B expression with RNA interference potentiated insulin secretion both in rat islets and insulinoma cells and contributed to a 2–3.5-fold increase in glucose-induced insulin release [41]. In contrast to the abolition of pulsatile glucose-dependent insulin secretion in knockdown cells, an overall increase in the mean value of the secreted insulin was observed [39]. It was also confirmed that PDE8B has been essential for the signaling pattern of [cAMP]_pm_ in β cells and is disrupted when its expression is suppressed [9]. Other studies have indicated PDE8 selective inhibition (PF-04957325 and PF-04671536) did not regulate resting cAMP levels in both INS-1 cells and primary human β-cells. It was also shown that there was no significant PDE8B inhibitory effect on GSIS in both INS cells and human islets [13].

### 3.9. The PDE9 Family

The PDE9 family is known to have the highest affinity for cGMP of any PDE [87,88]. It is well established that PDE9A is encoded by a single gene and expressed in more than 20 isoforms derived from alternative splicing of mRNA [89]. It was recognized that PDE9 is located in the brain, spleen, kidneys, and various gastrointestinal tissues [90], including in the pancreas [9]. The location of the family of individual PDE9 enzymes has been recognized in the cytosol (PDE9A5) but also reported that one is in the nucleus (PDE9A1) [44]. The inhibitors of PDE9A are in clinical development for Alzheimer’s disease [91] and as a potential therapeutic for diabetes [24]. Most of the designed inhibitors have been tested in preclinical and clinical trials as potential drugs for Alzheimer’s disease [90], although it is known for sure that the PDE9 is insensitive to the inhibition of IBMX [92]. To our knowledge, there are no studies showing that a selective PDE9 inhibitor affects GSIS in β cells.

### 3.10. The PDE10 Family

The PDE10 family hydrolyses both cAMP and cGMP but has a higher affinity for cAMP [93]. PDE10 is encoded by a single gene that gives rise to 18 splice variants [40]. The enzyme has been recognized to contain a tandem of GAF regulatory domains in the N-terminal region, and binding of cAMP to GAF-B increases enzyme activation at least 3-fold [93]. The expression was recognized in the brain (mainly striatum) and the peripheral tissues (testis and pancreas) [94]. Additional to the above data, the presence of PDE10A was confirmed in the pancreas [9,26], mouse β-Min6 cells, and human HP62 cultured cells. Several studies have shown that inhibition of PDE10A expression increases the insulinotropic effect in response to glucose from the rat pancreatic islets [95]. Of all the compounds tested, selective PDE10A inhibitors have shown that the quinoline subclasses (incubation with 8-mM glucose and forskolin) have been found to induce GSIS in vitro [96]. It is also worth noting that pharmacological inhibition of PDE10A protects mice against diet-induced obesity and insulin resistance, thus presenting the potential benefits of treatment with PDE10A inhibitors [94].

### 3.11. The PDE11 Family

The PDE11 family hydrolyses both cAMP and cGMP, and it is the last PDE family discovered [97]. In humans the PDE11A gene (chromosome 2q31.2) encodes four isoforms [19,40]. It has been shown to contain only one GAF domain [98]. The PDE11 isoform was detected in prostate, skeletal muscle, pituitary gland, liver, heart [99], and is significantly expressed in the pancreatic islet cell, suggesting it is involved in modulating synthesis and glucose-stimulated insulin secretion [41,100].

## 4. The Potential Role of PDEs Inhibition in Regeneration and Diabetes Mellitus (Type 1 and 2)

There are many strategies to support diabetes in the function of pancreatic β cells in a cAMP-dependent manner. Popular oral drugs used in the treatment of type 2 diabetes (sulfonylureas, thiazolidinediones), as well as human insulin preparations (for both types), cause weight gain, which are consequently unfavorable for the metabolic control profile and increase the risk of cardiovascular diseases, often despite a reduction in glycated hemoglobin (HbA1c) [101]. New therapeutic assumptions for type 1 of diabetes guaranteeing the protection and regeneration of endogenous islets or based on the treatment of primary autoimmunity through selective modulation of essential immune cells may exclude the current standard treatment limited to regimen glycemic control and force systematic injections of endogenous insulin. On the other hand, it is also promising to focus on treating autoimmunity that causes the disease and to design a strategy to delay or prevent the onset of this disease [102]. In contrast, for type 2 diabetes, incretin hormone therapy plays an important role. These factors increased intracellular cAMP in β cells by acting through G protein-coupled receptors in the β cell membrane. The GLP-1 reacts with Gsα through it acts on adenylyl cyclase and cAMP [103]. GLP-1 acts in two ways: through cAMP effectors mechanism, increasing insulin synthesis, supporting β-cell proliferation and inhibiting β-cell apoptosis, or in a second way, activating cAMP-independent pathways [104,105,106]. However, the use of these factors does not provide complete protection against the development of diabetes. The other disadvantages of endogenous insulin secretagogues in the treatment of type 2 diabetes are also their peptidyl nature, which requires administration by injection. Their action is short-lived due to the short half-life of the peptides. Nevertheless, basic studies have shown that both selective and non-selective inhibition of cyclic nucleotide phosphodiesterases may be important for GLP-1 secretion and enhance its action [107]. Thus, the recognition of insulin secretion modulators, maintenance of function dynamics, growth, survival, and β cell activity will be an important step on the long road to our ultimate goal of glycemic control and homeostasis in diabetes. New therapeutic strategies are currently being investigated to increase the selectivity and specificity with which PDEs are targeted. Interest in pharmacological agents aimed at improving the function of β cells and maintaining homeostasis in diabetes may prove to be the most effective method for its treatment [26,41,108]. The intracellular concentration of cAMP has a beneficially important role for β cell replication, survival, and insulin secretion [109,110]. It has been shown that cAMP may participate in a protective mechanism against NO-mediated impairment of β cell function [62]. Nevertheless, it should be mentioned that inhibition of, e.g., PDE3 and PDE4 in the liver will result in increased gluconeogenesis [76] (phosphodiesterase activity contributes to the reduction in glucose release from the liver). As a result, it can cause hyperglycemia in diabetic patients [110]. Gluconeogenesis in the liver is initiated by glucagon, which is responsible for its increase and the production of fasting glucose [111]. Metformin, used to treat diabetes, reduces hepatic glucose production and triggers the action of AMP-activated protein kinase (AMPK) [110,112,113]. AMPK has been shown to antagonize the effects of glucagon by activating PDE4B (mice) and lower cAMP levels and PKA activation [111]. The inhibition of phosphodiesterases is likely to be effective in the case of insulin resistance and β cell dysfunction, but, unfortunately, it may cause undesirable effects in the process of regulating gluconeogenesis.

PDE inhibitors selected in basic research do not always meet the expectations of their performance in preclinical and clinical trials. In addition, the matter is not simplified by the awareness and observation of side effects that manifest themselves in a longer time of exposure. Nevertheless, some selective PDE inhibitors have been shown to be therapeutically successful, and as a result, have been approved by the FDA and entered the market.

## 5. Regeneration

Basic research in animal models suggests that inhibition of individual PDEs may lead to β cell regeneration or replication. It has been identified that cilostamide and zardaverine could augment the number of zebrafish β cells vis the adenosine pathway and promote β cell regeneration. Whereas the adenosine deaminase inhibitor and the PDE2 inhibitor (EHNA) also increased the regeneration of β cells [114]. Moreover, stimulation of β cell replication in vitro, induce by IBMX, was observed [115]. The therapeutic utility of nonselective PDEs is limited, among others, by the induction of hepatic glucose production and hyperglycemia [116]. The research carried out on pancreatic islet rats identified the ability of PDEs to promote β cell replication (markers: ki67, insulin promoter factor 1 (PDX-1), proliferating cell nuclear antigen (PCNA)) by zardaverine, trequesin (the highest value of about 6-fold was obtained), and cilostamide. Moreover, cilostamide, cilostazol, and milrinone stimulated β-cell replication by increasing intracellular cAMP. According to Zhao et al. [115], the Food and Drug Administration (FDA) approved the above drugs.

## 6. Type 1 Diabetes Mellitus

Type 1 diabetes mellitus is caused by a chronic cell-mediated autoimmune disease characterized by selective destruction of β cells mediated by macrophages, lymphocytes T, and autoantibodies [117,118]. The development of type 1 diabetes also largely depends on proinflammatory cytokines (IL-1, tumor necrosis factor (TNF), interferon (IFN)) are also mediators of islet β cell dysfunction and apoptosis [119]. Cytokines initiate the activation of a gene network controlled by transcription factors, inducing the formation of nitric oxide (NO) and chemokines, followed by the activation of mitogen-activated protein kinases, which, in consequence, cause cell apoptosis [120].

PDE inhibitors not only increase intracellular cAMP/cGMP concentrations, they also show therapeutic efficacy in the treatment of autoimmune and/or inflammatory diseases [121,122,123,124,125]. In vitro studies have shown that increasing the concentration of cAMP by adding 8-Br-cAMP or dibutyryl-cAMP reduces the streptozotocin (STZ)-induced mortality of MIN6N8 cells. Moreover, molecular studies on these cells revealed an increase in PDE3A but not PDE3B expression. Incubation with cilostazol, a selective PDE3 inhibitor, significantly reduced the death of these cells, thus confirming the significant protective role of cAMP in type 1 diabetes. Similar results were obtained in studies carried out in an animal model (Mt3^+/+^ mice) with STZ induced diabetes, where PDE3A was shown to be implicated in apoptosis of pancreatic islet cells in these animals, and the use of cilostazol alleviated diabetes [126]. Studies in mice treated with PTX experimentally induced by SZT diabetes showed a reduction in blood glucose levels, increased plasma insulin concentration, and inhibition of T-cell proliferation. Moreover, increased IL-10 production in the spleen culture supernatant and decrease in Th1 and Th17 cytokines level (IFN-γ and IL-17) in PTX-treated mice was observed [127]. Rolipram, a selective PDE4 inhibitor, and pentoxifylline, which is a general inhibitor and also blocks the adenosine receptor, have been shown to increase intracellular cAMP levels and may thus suppress inflammatory cytokine production, preventing idiopathic diabetes and cyclophosphamide-accelerated diabetes (CYP) in nondiabetic (NOD) and suggest that the PDE inhibitors (cilostamide, rolipram, and PTX) can protect islets against autoimmunity [18]. Both rolipram and PTX administered i.p., twice a day, were shown to have protective effects against type 1 diabetes in NOD mice. The incidence was observed to be three to four times smaller than in untreated animals. The protection of NOD mice was maintained for 10 weeks after the cessation of the drug treatment. Immunochemistry indicated preserved insulin-producing cells. Nevertheless, PDE inhibitors have been shown to have anti-inflammatory or immunosuppressive effects, and symptoms of diabetes slowly return upon discontinuation [128,129]. PTX administered in vivo to STZ-induced diabetic animals CBA/H mice and DA rats (Dark Agouti rats), significantly reduced hyperglycemia. The protective effect of the inhibitor was shown to last for at least 8 weeks after the end of the experiment [130]. PDE inhibitors (rolipram and PTX), by generating an increase in cAMP, affect the changes in the production of pro-inflammatory cytokines. Moreover, their effect is a significantly reduced transcription and translation of TNFα, and reduced production of IFNγ and IL-12 [18,128]. There were significant differences in serum cGMP levels in rats with type 1 diabetes after treatment with vardenafil compared to control diabetic animals. In addition, there was a slight increase in serum glucose and a poor decrease in body weight in animals treated with a PDE5 inhibitor compared to the control group with type 1 diabetes [131]. PTX has been shown to lower blood glucose levels in animals with STZ-induced diabetes and revealed anti-hyperglycemic properties. Interestingly, it may also increase serum insulin levels in mice with type 1 diabetes [127]. PTX inhibits inflammatory cytokines TNFα, IFNy, and IL-12. It has also been shown to be effective in treating inflammatory and autoimmune diseases [128,132,133]. It has been shown in clinical trials with reduced insulin requirements in children with recent diabetes. A small group of children with newly diagnosed diabetes and treated with insulin also received PTX (1200–2400 mg/day). The results clearly showed that the administration of PDX, for a period of 3–12 months, decreased the daily dose of insulin and lengthened the “honeymoon” compared to the control group. Nevertheless, it proved ineffective on the issue of preventing the illness [134]. Long-term treatment with PTX did not cause any significant changes in blood glucose and HbA1 levels between the patients with type 1 or type 2 diabetes [135]. Clinical trials conducted on both types of diabetics, concerning PDE5 inhibitors usually focus their attention on erectile function and do not refer to the effects of changes in diabetes or glycaemia after therapy practically at all [136,137].

## 7. Diabetes Type 2

Obesity and insulin resistance are associated with type 2 diabetes [138]. Insulin resistance or impairment of insulin sensitivity has both a genetic and an environmental background [139]. A hallmark of insulin resistance is a reduced ability of a physiological insulin concentration to stimulate muscle glucose uptake [140,141]. As a consequence, chronic insulin resistance leads to **lower** function, β cell mass (mainly by apoptosis), and progress of diabetes type 2 [141,142]. Elevated fasting plasma insulin is also the cause of other pathological conditions: lipodystrophy and non-alcoholic fatty liver disease, etc. [139,143]. Hyperglycaemia and hyperlipidemia contribute to the overproduction of reactive oxygen species (ROS) and reactive forms of nitrogen (RNS), which, in turn, causes oxidative stress [144]. Pancreatic β cells show low endogenous antioxidant capacity [145], and increased ROS production in these cells results in their dysfunction. Triglyceride and fatty acid induced synthesis of ceramides in hyperlipidemia, and in combination with excessive production of nitric oxide (NO), leads to β-cell apoptosis. Due to the broader range of action resulting from the presence of PDE in various tissues, it has been observed that their inhibitors have pleiotropic effects and offer broad prophylaxis in relation to diabetes mellitus and its accompanying diseases.

In pancreatic islets from patients with type 2 diabetes, an increased expression of PDE3A (8-fold) and PDE3B (3-fold) was observed compared to the results obtained from non-diabetic pancreatic islets. Moreover, a low concentration of cAMP has been demonstrated after incubation with high glucose [62]. On this basis, it can be concluded that inhibition of PDEs can bring many benefits in the treatment of diabetes. Compared to non-specific PDE inhibitors, such as theophylline, caffeine, or IBMX, it can be concluded that PDE inhibitors specific for the enzyme subclass will have a better benefit-risk ratio. Commercially available drugs and most recognized PDE3 inhibitors, cilostazol, and milrinone, have a wide range of benefits resulting from their activity. Unfortunately, their long-term action has many undesirable side effects. Cilostazol has wide pharmacological applications, vasodilation, inhibition of platelet activation and aggregation, increased blood flow to the extremities, and inhibition of cell growth in vascular smooth muscles [146]. In addition, it have been shown that it affects the lipid metabolism, reducing the concentration of triglycerides, chylomicrons, and lipoproteins alternatively (VLDL, very low-density lipoprotein), which supports the anti-aggregation effect [147,148]. Previous studies carried out on animal models with type 2 diabetes demonstrated that the inhibition of PDE3 by cilostazol revised insulin sensitivity by stimulating the peroxisome proliferator-activated receptor (PPAR) γ transcription and anti-inflammatory effects of cAMP-depends in a db/db mouse model of type 2 diabetes [149]. In subsequent studies, cilostazol treatment for four weeks, improved insulin resistance in STZ-induced non-insulin-dependent diabetic rats was indicated [150]. Moreover, the reduction in insulin resistance and/or abdominal fat accumulation in an animal model of type 2 diabetes was observed [151]. Other studies carried out on STZ-NA-induced diabetic rats showed a decreased concentration of cAMP, although the inhibition of PDE3 (amrinone) was insufficient to improve insulin secretion [60]. Daily administration of cilostazol to db/db mice significantly improved glucose tolerance and insulin sensitivity in a dose-dependent manner. Moreover, a reduced size of adipocytes was observed and suppressed the expression of tumor necrosis factor α (TNFα) in epididymal adipose tissue in these mice [152]. In studies conducted on human pancreatic islets in vitro, cilostazol significantly potentiated GSIS, and its effect was stronger in diabetic islets than in non-diabetic islets [62]. The selective PDE3 inhibitors were originally considered as candidates for type 2 diabetes mellitus. Nevertheless, the presented results of clinical trials do not give unequivocal results. In several clinical trials, the effect of cilostazol resulted in improvement in peripheral blood flow and increased insulin sensitivity by reducing the inflammatory process [146,153,154]. Moreover, an improvement in glycemic control and reduced insulin resistance, and decreased hemoglobin A1c (HbA1c) level in cilostazol groups was observed [123,146]. Cilostazol may lead to an increase in blood glucose, possibly by overproducing glucose in the liver in a way that exceeds insulin’s ability to lower it [153]. By contrast, other clinical trials have not achieved changes in the body mass index, HbA1c, fasting glucose, or changes in blood glucose [155,156]. The FDA has approved cilastozol for the treatment of intermittent claudication. Cilostazol is still being tested clinically as a potential drug for atherosclerotic episodes in type 2 diabetes (NCT01252056, NCT00823849), type 2 antiplatelet aggregation (NCT03248401, NCT02983214, NCT02933788), Alzheimer’s disease (NCT01409564), and in other contexts as well [157,158]. However, for instance, the tests of type 2 diabetes peripheral neuropathy have been unsuccessful (Phase IV/completed 2009, NCT01076478) [156].

The PDE4 has regulatory properties in immune/inflammatory cells, and in the central nervous system, their selective inhibition exerts a strong anti-inflammatory effect [73], thermogenic function, and neuroendocrine function [159]. Originally, the function of PDE4 inhibitors was mainly associated with inflammatory diseases, including asthma and chronic obstructive pulmonary disease (COPD) [121]. Basic research has shown a significant role of PDE4 in the regulation of GLP-1 homeostasis, which turned out to be an additional advantage of using PDE4 inhibitors in the treatment of type 2 diabetes [107]. Preclinical studies revealed that selective inhibition of PDE4 TAK-648 increased glucose tolerance and HbA1c and improved pancreatic islet morphology in db/db mice, and additionally caused reductions in food and water consumption and slight weight loss in these animals [160]. Otherwise, PDE4B-deficient mice show reduced adiposity and decreased high-fat diet-induced adipose inflammation. TNF-mRNA levels and macrophage infiltration were reduced [161]. The PDE4 inhibitor rolipram protects mice on a high-fat diet against obesity and glucose intolerance [162]. Moreover, antidiabetic activity was demonstrate through the participation of PDE4 in the metabolism of glucose and lipids [163]. Few of the PDE4 inhibitors are under clinical evaluation for the treatment of disorders such as type 2 diabetes and non-alcoholic steatohepatitis. Clinical trial results show that roflumilast improves glucose homeostasis in previously untreated patients with newly diagnosis diabetes type 2. In addition, the effect of roflumilast (12 weeks) in patients reduced free fatty acids, fasting plasma glucose, glycerol and glucagon levels and resulted in slightly higher insulin and C-peptide levels compared to patients in the placebo group [164]. In addition, a slight loss of body weight has been reported in various clinical trials in the roflumilast-treated patient groups [164,165,166]. The influence of roflumilast on glucose homeostasis shows activity similar to the activators of the GLP-1 receptor, incretin mimetics [164], confirms the earlier observations of basic research [107]. The first phase of clinical trials (NCT02480439, NCT02684396, NCT02430870) regarding the possibility of using the PDE4 inhibitor-TAK-648/Takeda in type 2 diabetes were completed in 2015.

PDE5 inhibitors, class II drugs, lead to an increase in cGMP. The main substances that inhibit PDE5 activity and are commercially available as drug agents are sildenafil, vardenafil, tadalafil, and the recently approved avanafil [125]. Sildenafil was the first selective PDE5 inhibitor approved for the treatment of erectile dysfunction in 1998 [167], followed by vardenafil and tadalafil, which were released in 2003 [91]. Preclinical and clinical studies have shown that PDE5 inhibition has a beneficial effect not only on improving β cell function [168] but also on increasing insulin sensitivity of other peripheral tissues (skeletal muscle cells, adipocytes, and endothelial cells), thus improving insulin resistance [169,170,171]. However, clinical trials of type 2 diabetes based on the effects of Vardenafil (Levitra)/Bayer/GlaxoSmithKline showed no efficacy (Phase II/completed 2014, NCT02219646) [172]. Chronic inhibition of PDE5 has also been shown to improve fibrinolytic balance and albuminuria in people with prediabetes [168]. The participation of PDE5 in numerous physiological functions via the NO-cGMP pathway has been confirmed, which is directly related to its expression in various human tissues (brain, lung, heart, liver, kidney, and skeletal muscles) [173]. Moreover, PDE5 and nitric oxide synthase (NOS) have also been shown to be highly expressed in a variety of tissues commonly associated with the regulation of glucose metabolism [174,175]. Additionally, it may favorably influence glucose metabolism in type 2 diabetes, mediating increased glucose uptake by skeletal muscle and reducing insulin resistance [20,81,141,176]. It has been found that an increase in cGMP, in pancreatic islets, intensifies glucose-stimulated insulin secretion. However, constant, long-term stimulation causes the opposite effect [177]. It was shown that a single dose of tadalafil increases the sensitivity to insulin in cells of skeletal muscle by promoting glucose transporter 4 (GLUT4) gene expression and translocation to the cell membrane [178]. Moreover, it also induced the activation of both the peroxisome proliferator-activated gamma receptor (PPARγ) and the insulin receptor substrate (IRS-1) and stimulates the phosphorylation of both IRS-1, PKB, and glycogen synthase 3 beta kinase (GSK3β) [169]. However, the full mechanism of the influence of PDE5 inhibitors on glucose homeostasis has not yet been fully elucidated [179]. In part, this effect may also be due to the prevention of endothelial dysfunction [180] or systemic inflammation [174]. In addition, in patients with type 2 diabetes, chronic PDE5 inhibition with sildenafil lowered the biomarker associated with vascular damage and improved glycometabolic control [180]. Clinical trials have shown that PDE5 inhibitors (sildenafil, PF-00489791) may have an effect on glucose metabolism and have important implications for the treatment of insulin resistance in diabetes and obesity. It has been proven that daily therapy with them for a period of 1–4 months resulted in a significant reduction in mean HbA1c (about 0.3–0.59%) compared to the baseline value [181,182], and the reduction in hyperglycemia was comparable to effects of oral antidiabetic drugs administered over a longer period of treatment [183]. Tadalafil as an inhibitor of PDE5 enhanced cGMP-dependant insulin secretion and improved β cell function [20], also in the metabolic syndrome [141], but no overall improvement in insulin resistance was observed [20]. In postmenopausal type 2 diabetic women, improvements in metabolic parameters as a result of tadalafil treatment have been observed. Studies have shown in these patients a lower glucose permeability surface area and improved metabolism of muscle and fat tissue [184]. While studies were carried out on obese men (insulin secretion/sensitivity in obesity (NCT02595684, phase IV/completed 2015) demonstrated that tadalafil administration for 28 days did not modify insulin secretion or insulin sensitivity [185]. In other clinical trials, the role of tadalafil (Cialis or Adcirca)/Eli Lilly was determined in context of type 2 diabetes (postprandial hyperglycaemia) (NCT01238224, phase I/terminated early 2011), obesity (NCT0255404, phase IV/completed 2015), insulin resistance in type 2 diabetes (NCT02601989, phase II), diabetic cardiomyopathy (NCT01803828, phase IV) [170,186,187], whereas sildenafil caused an increase in peripheral insulin sensitivity to insulin in patients with pre-diabetes after 3 months of treatment [168]. However, in patients receiving sildenafil (100 mg/d), higher expression of miR-22-3p proteins (associated with myocardial hypertrophy and its remodeling) and sirtuin 1 (SIRT1) (its overexpression reduces insulin resistance) was demonstrated [188]. Nevertheless, the effectiveness of PDE5 inhibition may be limited under conditions of low NO production and decreased cGMP concentration, as found in obese and diabetic patients, and may not reach the appropriate cGMP threshold for treatment effectiveness [91]. Clinical trials indicate a significant potential of PDE5 to modulate metabolic complications related to diabetes mellitus and with appropriate prevention and management of macrovascular and microvascular complications [172,173,180,189]. It has also been shown that diabetic patients are more likely to develop heart diseases than they are patients without diabetes [190]. These observations are supported by numerous animal studies that have confirmed that inhibition of PDE5 has a significant protective effect against myocardial damage/reperfusion (I/R), ischemic and diabetic cardiomyopathy, and myocardial hypertrophy [191]. Arguably, cardiac adrenergic signaling interacts with PDEs activity (levels of cAMP and cGMP in cardiomyocytes), but PDE5, in particular, may have a significant effect on diabetic cardiomyopathy, including reduced cardiac inotropy. Changes in adrenergic modulation were observed in the heart of mice as a result of PDE5 inhibition. The β3 adrenergic receptor has also been shown to be associated with this effect, resulting in a negative inotropic response. Besides, the participation of the β2 adrenergic receptor in the development of diabetic cardiomyopathy has also been confirmed. The increased expression of phosphodiesterases in diabetic cardiomyocytes and a decrease in PKA activity were observed [190]. In animal models of experimentally induced diabetes or established cardiomyopathy, vardenafil improved heart function by activating nitric oxide synthase cGMP-PKG [192,193]. Moreover, sildenafil has been shown to be effective in reversing symptoms in murine models of heart failure [194,195,196]. Clinical trials in people with diabetes also show promising results. The inhibition of PDE5 revealed protective properties in heart failure, infarction, ventricular arrhythmia, ischemic disease/reperfusion injury [197]. While, in other studies, it was reported that sildenafil significantly decreased the elevated serum IL-8 levels associated with diabetic cardiomyopathy. Its effect was possibly related to transcriptional and posttranscriptional regulation [198]. In addition, it was found that sildenafil administered to patients in diabetic cardiomyopathy significantly decreased the level of Th1 CXCL10 chemokine in both blood and human cardiomyocytes [197]. Clinical trials of sildenafil (or Revatio)/Pfizer activity were also analyzed in the context of diabetic cardiomyopathy in type 2 diabetes. Testing was carried out at phase IV level and was completed in 2009 [170,188,197].

Endothelial dysfunction is the result of neglect of glucose control, and endothelial-dependent vasodilation may manifest itself in the early stages of type 2 diabetes [199]. Moreover, it is suspected that inhibition of PDE1B, −9A, −10A, or −11A may increase the efficiency of microvascular perfusion and sensitize, e.g., muscles to the presence of insulin. Animal studies have shown that a coinfusion of zaprinast with insulin increases insulin sensitivity and is manifested by an increase in muscle glucose uptake [200]. Selective PDE9 inhibitors have also been explored as potential therapeutic agents for the treatment of diabetes and other diseases, such as the central nervous system, including Alzheimer’s [201,202]. However, there are no studies available for type 2 diabetes. The situation is similar in the case of PDE10 and −11. Clinical trials for PDE9 and PDE10 with the use of their selective inhibitors have been conducted since 2013 and concern only schizophrenia and neurodegenerative diseases. However, in the case of PDE11, there are no such studies yet.

Phosphodiesterases have a wide range of applications in diabetes, and their versatility continues to surprise. Completing the knowledge about them will certainly not only facilitate effective targeting of PDEs but also avoid side effects to fully use their potential as therapeutic targets in diabetes.

## Abbreviations

AC	Adenylyl cyclase
ADP	Adenosine diphosphate
AKAP	A-kinase anchoring proteins
AMPK	AMP-activated protein kinase
ATP	Adenosine triphosphate
BMI	Body mass index
CAM	Calmodulin
[Ca^2+^]_i_	Intracellular calcium
cAMP	Cyclic AMP
[cAMP]_i_	Intracellular camp
[cAMP]pm	cAMP in the sub-plasma-membrane space
cGMP	Cyclic GMP
CYP	Cyclophosphamide
DB-cAMP	Dibutyryl camp
EC	Endothelial cell
EPAC	Exchange protein directly activated by camp
ERK	Extracellular signal-regulated kinases
FDA	Food and Drug Administration
GAD	Glutamic acid decarboxylase
GAF	Guanylate cyclase
GC	G protein coupled receptors
GCPRs	G protein-coupled receptors
GDP	Guanosine diphosphate
GLP-1	Glucagon-like peptide-1
GLUT	Glucose transporter
GSIS	Glucose- stimulated insulin secretion
GSK3	Glycogen synthase kinase
GTP	Guanosine-5’-triphosphate
Hba1C	Glycated hemoglobin
IGF	Insulin-like growth factor
IFN	Interferon
IL	Interleukin
IRS	Insulin receptor substrate
K_ATP_	Channels, ATP-sensitive K^+^ channels
NO	Nitric oxide
NOD	Nondiabetic
PAS	Per-arnt-sim domain
PCNA	Proliferating cell nuclear antigen
PDE	Phosphodiesterase
PDX-1	Insulin promoter factor 1
RNS	Reactive forms of nitrogen
ROS	Reactive oxygen species
p42^MAPK^	Mitogen-activated protein kinases
PIK3	Phosphatidylinositol 3-kinase
PKA	Protein kinase A
PKB	Protein kinase B
PKG	Protein kinase G
PPAR	Peroxisome proliferator-activated receptors
SIRT1	Sirtuin 1
STZ	Streptozotocin
T2D	Type 2 diabetes
TNF	Tumor necrosis factor
UCR	Up-stream conserved region
VGCC	Voltage-gated Ca^2+^ channel
VLDL	Very-low-density lipoprotein
WHO	World health organization
